# 505. Non-recreational Burn Injuries are Associated with Worse Pain and Social Integration

**DOI:** 10.1093/jbcr/irag033.164

**Published:** 2026-04-06

**Authors:** Sarah Wang, Bill Insko, Kara McMullen, Caitlin M Orton, Karen J Kowalske, Juan P Herrera-Escobar, Haig A Yenikomshian

**Affiliations:** Keck School of Medicine, University of Southern California, Los Angeles, CA; Department of Rehabilitation Medicine, University of Washington, Seattle, WA; Department of Rehabilitation Medicine, University of Washington, Seattle, WA; Department of Surgery, University of Washington Medicine Regional Burn Center, Harborview Medical Center, Seattle, WA; Department of Physical Medicine and Rehabilitation, University of Texas Southwest Medical Center, Dallas, TX; Department of Surgery, Brigham and Women's Hospital, Harvard Medical School, Boston, MA; Division of Plastic and Reconstructive Surgery, Keck School of Medicine, University of Southern California, Los Angeles, CA

## Abstract

**Introduction:**

While established predictors such as burn severity and pre-injury employment influence return-to-work (RTW) outcomes, the specific impact of injury circumstance— where and how the burn occurred— remains less clear. Different environments in which a burn happens, whether at work, during a recreational activity, or at home, are associated with unique recovery trajectories and psychosocial challenges. This study investigates the impact of burn injury circumstance on RTW and psychosocial outcomes in adult burn survivors to identify at-risk populations that would benefit from tailored support measures.

**Methods:**

Data was collected from adult burn survivors enrolled in a national longitudinal database from 2015 to 2024. Participants were categorized by injury circumstance: work-related (WR), non-work and non-recreational related (NWR), and recreational (Rec). Only participants who were employed at the time of injury were included. The primary outcomes were employment status and patient-reported outcomes in pain, anxiety, depression, fatigue, physical function, and social reintegration at 12 months. Multivariable logistic and linear regression models were used to assess the association between circumstance and outcomes while adjusting for burn size (%TBSA), inhalation injury, and amputations. The significance level was set at *p*<.05.

**Results:**

Among 2117 participants, 735 (34.7%) were in the WR cohort, 1148 (54.2%) in the NWR cohort, and 234 (11.1%) in the Rec cohort. By the 12-month follow up, the majority of patients were employed in all three cohorts. After adjusting for confounders, there was no difference in employment rates at 12 months when comparing Rec to WR (OR = 0.91, 95% CI = 0.50 – 1.66, *p*=.62) or NWR (OR = 1.02, 95% CI = 0.57 – 1.83, *p*=.73) groups. Greater %TBSA, presence of inhalation injury, and amputations were significantly associated with reduced odds of employment. Compared to Rec injuries, WR injuries were associated with increased pain interference (PROMIS Pain Interference, Rec: 49.0 vs. WR: 53.1, *p*=.02), and both WR and NWR cohorts had more difficulty with social reintegration (PROMIS Ability to Participate in Social Roles, Rec: 55.1 vs. WR: 51.3, *p*=.03; NWR: 51.4, *p*=.02). No significant differences were observed with anxiety, depression, fatigue, or physical function scores.

**Conclusions:**

This study highlights that the burn injury circumstance has a distinct association with pain and social reintegration, independent of injury severity. Predicting successful RTW remains challenging, as outcomes are often dependent on other contextual factors as well, such as the patient's financial need, enjoyment of their work, and the availability of vocational resources.

**Applicability of Research to Practice:**

Individuals with non-recreational burns may benefit from targeted interventions for pain management and social reintegration, regardless of injury severity.

**Funding for the study:**

The contents of this abstract were developed under a grant from the National Institute on Disability, Independent Living, and Rehabilitation Research (NIDILRR grant number 90DPBU0007). NIDILRR is a Center within the Administration for Community Living (ACL), Department of health and Human Services (HHS). The contents of this abstract do not necessarily represent the policy of NIDILRR, ACL, or HHS, and you should not assume endorsement by the Federal Government.

